# Opioid-Induced Hyperalgesia in a Cancer Patient on High-Dose Methadone Maintenance Therapy: A Case for Subspecialty Opioid Use Disorder Primary Care

**DOI:** 10.7759/cureus.12345

**Published:** 2020-12-28

**Authors:** Ebubechukwu Ezeh, Davinder Singh, Varun Dobariya, Esiemoghie J Akhigbe, Christine Gilkerson

**Affiliations:** 1 Internal Medicine, Marshall University, Joan C. Edwards School of Medicine, Huntington, USA

**Keywords:** opioid-induced hyperalgesia, high-dose methadone maintenance therapy, opioid use disorder

## Abstract

As opioid use disorder (OUD) reaches epidemic levels in the United States, medication-assisted treatment (MAT) plays a central role in its treatment. Methadone, a long-acting mu-opioid receptor agonist has been shown to be effective in managing OUD. It is also known that chronic opioid therapy may have the paradoxical effect of increased sensitivity to pain, a phenomenon called opioid-induced hyperalgesia (OIH). This presents a conundrum when a patient such as ours, on MAT presents with acute pain and OIH. This case report illustrates the current challenges health care providers encounter when treating patients on chronic MAT for non-opioid-related conditions. As this patient population ages, these encounters will become more common. These patients will need appropriate health care screening and chronic care management. This case serves two purposes; to highlight the difficulty in treating acute pain in patients on long-term high-dose methadone coupled with the missed opportunity for primary care for OUD patient population, and proposes that education reforms in this area be implemented now.

## Introduction

Opioid use disorder (OUD) has become an epidemic in the United States, and as such, medication-assisted treatment (MAT) has become an integral part of health care delivery. MAT has been found to reduce morbidity and mortality, decrease overdose deaths, reduce transmission of infectious disease, increase treatment retention, improve social functioning, and reduce criminal activity [1]. Thus, the number of federally approved Opioid Treatment Programs (OTPs) offering MAT has increased accordingly. For instance, the number of OTPs increased from approximately 1,100 in 2003 to almost 1,500 by the end of 2016 [2]. Methadone, a long-acting mu-opioid receptor agonist in use since 1964 for the treatment of OUD, may be dispensed in these OTPs. According to the Substance Abuse and Mental Health Services Administration, the number of clients receiving methadone increased from about 227,000 in 2003 to over 350,000 in 2015 [2]. Clients receiving treatment with methadone accounted for approximately 21% to 25% of all substance abuse treatment clients each year. The increase in the number of clients receiving methadone treatment coupled with the stability of the proportion of clients receiving this treatment indicates that the overall availability of methadone treatment has increased over time [2]. The implication of this is that clinicians will continue to encounter more patients who are on MAT, who will also need primary care screening and chronic care management that is currently lacking.

When used in OUD, methadone prevents withdrawal symptoms for 24 hours or longer, decreases cravings for opioids, and decreases the euphoria associated with illicit opioid use by maintaining high levels of opioid tolerance [3]. Many individuals receiving methadone maintenance treatment (MMT) for opioid addiction may also require treatment for acute or chronic pain. However, effective pain management in this patient population is complicated by many factors, including heightened pain sensitivity, high opioid tolerance, and variable cross-tolerance to opioid pain medications. The phenomenon of heightened pain sensitivity referred to as opioid-induced hyperalgesia (OIH) presents a conundrum between controlling the acute pain of a cancer patient on MMT and managing OIH [4,5]. As this patient population ages, unique challenges will accompany their health care needs for non-opioid use-related conditions as described in our case. Thus, clinicians will need to be educated on best practices when they do encounter this patient population.

## Case presentation

A 50-year-old male with a past medical history significant for chronic hepatitis C and opioid use disorder on MMT for 12 years was presented with a six-week history of left hip pain, left shoulder pain, and unintentional weight loss of over 25 pounds in two months. He also reported a productive cough with whitish sputum, shortness of breath, chest pain, and fatigue. He was on a daily dose of methadone 120 mg for the past 12 years for OUD. Physical examination findings showed a cachectic man with temporal wasting, evidence of dehydration, and a palpable right-sided abdominal mass. Initial labs were remarkable for marked hypercalcemia and findings suggestive of prerenal acute kidney injury (AKI). Imaging studies were remarkable for metastatic lung lesions, and multiple osteolytic lesions in the left femur, ribs, left humerus, C6, and T6 vertebrae. Abdominal imaging showed a large renal mass with renal vein thrombosis. The diagnosis of renal cell carcinoma as the primary malignancy was suspected. The prognosis was discussed with the patient, and he expressed his desire to move forward with treatment. The patient’s AKI improved with intravenous fluids. Hypercalcemia also improved after zoledronic acid treatment. However, the patient's reported pain score was constantly 9 out of 10. Attempts to reduce the dose of his MMT lead to opioid withdrawal symptoms. The pain management team was consulted, and a decision was made to split the patient’s total home methadone dose into 40 mg three times a day. Other non-opioid pain medications such as high-dose Tylenol and Toradol were added to his regimen but the patient reported little to no pain relief. Then, a short-acting opioid in the form of oxycodone 10 mg every four hours was added to control his breakthrough pain. However, the patient continued to have residual generalized body pain which he consistently rated 7 out of 10. His oxycodone was further increased to 20 mg which provided temporary pain relief. After a few days, his pain was still persistent and the decision was made to continue the oxycodone at 10 mg every 12 hours and add an extended-release form of oxycodone 10 mg to his methadone, Tylenol, and Toradol regimen.

It was at this point that the patient's pain score reduced to less than 4 out of 10 and he was discharged home on the new pain regimen. With this extensive course of narcotics, laxatives were added to prevent constipation. However, two weeks later, the patient presented complaining of generalized body pain. This time, oxycodone was increased from 10 mg to 45 mg, and finally, a Fentanyl patch of 25 mg every 72 hours was added to his overall pain regimen. The new regimen was able to provide sufficient pain relief.

## Discussion

This case illustrates the unique challenges of trying to achieve pain control in a cancer patient on long-term opioid replacement therapy for OUD. It is our position that clinicians will encounter these situations more and more as this patient population ages. Chronic high-dose methadone therapy as in this patient leads to the development of tolerance and consequently reduced responsiveness to the analgesic effects of opioids [5]. Also, pain studies have shown that MMT patients have hyperalgesia and that cross-tolerance to other opioids may be present, suggesting that they may need more analgesia than non-MMT patients [5]. OIH is another compounding factor that should be considered when increasing opioid doses fails to provide analgesic effects or when there is unexplainable pain exacerbation following opioid treatment [6].

The potential hyperalgesic state accompanying opioid dependence complicates pain management [7]. The major challenge is determining when and how to achieve incremental opioid dosing and determining when to stop, assuming there is an end date. The key to that decision is dependent on the goal of treatment. If the goal is pain relief, the general approach to acute pain management in patients who take methadone is similar to the approach used for patients who take stable doses of other opioids on a chronic basis: continuation of the baseline opioid, which we did in this patient, and use of nonopioid analgesic strategies supplemented with incremental opioid if necessary. Once the acute pain subsides, the additional opioid should then be tapered down [8]. If tapering is not feasible, additional opioids for acute pain in these patients should not be withheld for fear of worsening the OUD. As long as medications for acute pain are tapered promptly as the pain resolves, the patient's course of treatment for OUD can continue undisrupted [8].

Weaning a patient off methadone should be accomplished over months to years. That was not an option for our patient who has a new diagnosis of metastatic cancer. Also, methadone withdrawal signs must be anticipated and treated accordingly with other opioids during the weaning period. While we were eventually able to achieve pain control, the time to this was more prolonged as compared to someone with a similar clinical scenario who is not on chronic MMT. Given the advanced stage of the malignancy at presentation, it is conceivable that the high doses of methadone may have contributed to the patient not presenting earlier. Another factor that we feel contributed to this patient's late presentation is the lack of primary care involvement. He experienced progressive symptoms of weight loss, cough, and fatigue months prior to presentation without seeking any primary care attention. We feel periodic primary care assessment that coincided with this patient’s OUD treatment may have caught the disease process at a much earlier stage. The lack of available clinicians with expertise in this area contributed to the delay. In this regard, we propose that Continuing Medical Education (CME) training in this area be required for current practicing physicians. An example of such a training module is Providers' Clinical Support Services (PCSS). PCSS is a program funded by the Substance Abuse and Mental Health Services Administration (SAMHSA) and was created in response to the opioid overdose epidemic to train primary care providers in the evidence-based prevention and treatment of OUD and treatment of chronic pain. We further advocate for the incorporation of similar training modules into the Graduate Medical Education (GME) curriculum to better prepare future clinicians to care for the increasing number of patients needing care for non-OUD conditions who are on MAT.

## Conclusions

Because opioid cross-tolerance may be present, patients on MMT typically require high doses of opioids than opioid naïve patients. Undertreatment of acute pain is regarded as suboptimal medical management, and this patient population is at particularly high risk. Thus, pain should always be treated in these patients, irrespective of their MMT status. We were eventually able to achieve pain control; however, lack of expertise (both consultation and primary team) contributed to the delay. Clinicians will continue to encounter patients who are on chronic MAT for non-opioid use-related conditions such as our patient. A possible strategy to avoid this in the future could be the development of an OUD primary care specialty. Also, health care providers will need adequate training to better care for these patients. Integration of such training into the CME requirements and the General Medical Education (GME) curriculum would improve clinicians' knowledge about this pathology.
